# Colorectal Adenocarcinoma Derived From Mature Cystic Teratomas: A Case Report With Review of the Literature

**DOI:** 10.7759/cureus.44159

**Published:** 2023-08-26

**Authors:** Fnu Raja, Gopal Kumar, Azzam Hammad, Santhi Ganesan

**Affiliations:** 1 Pathology, MetroHealth Medical Center, Cleveland, USA

**Keywords:** immunohistochemistry, malignant transformation, colorectal adenocarcinoma, somatic malignancy, mature cystic teratoma

## Abstract

Mature cystic teratomas (MCTs) are the most common benign ovarian germ cell neoplasms in women of reproductive age. Rarely, somatic malignancies arise from MCTs, the most common being squamous cell carcinoma. Adenocarcinomas are less common and colorectal adenocarcinomas are extremely rare. We present a case of somatic adenocarcinoma of colorectal type which may pose challenges in diagnosis and treatment. A middle-aged female presented to the Emergency Department with lower abdominal pain. CT scan revealed an 11 cm sharply demarcated left pelvic mass. Laparoscopy showed a left ovarian mass with torsion, a smooth external surface, and thick brownish contents. An intraoperative evaluation was consistent with an adenocarcinoma. Permanent histopathology revealed adenocarcinoma of colorectal phenotype with necrosis. Additional evaluation of the cyst showed benign colonic epithelial lining. The immunohistochemistry (IHC) profile of positive CDX2 and CK20 and negative PAX8, CK7, ER, and PR suggested a colorectal-type somatic adenocarcinoma arising from the MCT and was staged as IA, after negative endoscopic findings. Due to their rarity and atypical symptoms, distinguishing metastatic tumors from MCT-derived somatic malignancies is a challenging process. CT scan and serum tumor markers can be helpful but are not definite. Thorough clinical evaluation and proper staging are necessary after pathologic evaluation. Extensive sampling and IHC can further characterize the origin of the tumor. Diligent sampling and a high index of suspicion in this case clinched the correct diagnosis and clinical management. The patient is being treated for stage IA ovarian cancer as opposed to stage IV metastatic colorectal cancer.

## Introduction

Mature cystic teratomas (MCTs), also known as dermoid cysts, are benign germ cell tumors composed of mature tissue derived from two or more embryonic cell layers (endoderm, mesoderm, or ectoderm). They account for 95% of all ovarian germ cell tumors, and 20% of all ovarian tumors [1]. MCTs commonly arise in reproductive-age women [2] and are mostly unilateral, while in 10% of cases, they can be bilateral [3]. The most accepted theory of their origin is from primordial germ cells [4].

Clinically, these are slow-growing tumors, and their presentation is quite variable. Approximately, 20% of the cases are clinically silent and found incidentally on radiologic work-up or during abdominopelvic surgeries for other reasons, but when they are enlarged, they can cause non-specific abdominopelvic pain, compressive effects on nearby organs, and can also present with acute abdomen when the cyst undergoes torsion or spontaneous rupture [3].

In a few cases, MCTs are associated with rare neurological autoimmune paraneoplastic encephalitis syndrome secondary to anti-N-methyl-D-aspartate receptor (NMDAR) antibodies. They are commonly seen in younger patients with a broad range of neurologic symptoms such as changes in behavior, memory loss to seizures, and autonomic instability [5]. The pathogenesis behind this process is the presence of immature neuronal tissue or nerves expressing the NR2 subunit of the NDMA receptor which reacts with antibodies [5]. Its prognosis depends mainly on the length of symptoms and other characteristics. If the teratoma is detected at an early stage, removal of the tumor is an effective treatment [5] while in later stages, plasmapheresis and immunosuppressive therapy are required in addition to surgery [6]. In 7% of the cases, this can lead to death [5].

## Case presentation

One of the rare and serious complications of MCTs is malignant transformation, seen in only 1-3 % of the cases [7]. Most of these cases are present in older or postmenopausal women [8], with the most common being squamous cell carcinoma (SCC), which accounts for approximately 85% of the cases [9]. Besides SCC, other rare malignancies such as basal cell carcinomas, melanomas, sarcomas, neuroectodermal, and adenocarcinomas have been reported as well. While adenocarcinomas account for up to 7% of cases [10], intestinal adenocarcinoma is one of the extremely rare events of malignant transformation. MCT-derived colorectal carcinoma is a rare entity and to the best of our knowledge, only 12 cases have been reported in the literature as summarized in Table 1. This entity is even rarer in the western hemisphere with only two cases being reported from the United States [11,12]. We are reporting the third case of somatic adenocarcinoma of the colorectal type which, due to its rarity, may pose challenges in diagnosis and treatment.

**Table 1:** Cases of intestinal adenocarcinoma arising from mature cystic teratomas reported in the literature

Case	Author	Reported Country	Age	Tumor size in largest dimension	FIGO stage
1	Fishman et al., (1998) [13]	Israel	38	20.0 cm	IIIC
2	Ueda et al., (2003) [14]	Japan	62	35.0 cm	IA
3	Kushima et al., (2004) [15]	Japan	52	6.4 cm	IA
4	Levine et al., (2004) [11]	United States	37	15.0 cm	IA
5	Güney et al., (2006) [16]	Turkey	38	NR	IA
6	Min et al., (2006) [17]	Korea	77	17.0 cm	IA
7	Takai et al., (2012) [18]	Japan	49	6.7 cm	IA
8	Hershkovitz et al., (2013) [19]	Israel	13	10.0 cm	IA
9	Li et al., (2014) [20]	China	51	5.8 cm	IA
10	Li et al, (2014) [20]	China	43	10.8 cm	IA
11	Clark et al., (2016) [12]	United States	42	17.0 cm	IA
12	Sunitha et al., (2017) [21]	India	33	21.0 cm	IA
13	Wan et al., (2019) [22]	Australia	58	10.0 cm	IA

A previously healthy postmenopausal woman presented to the Emergency Department for evaluation of non-specific lower abdominal pain. The pain was dull and intermittent, with radiation to the left flank without any relieving or exacerbating factors. It was not associated with nausea, vomiting, chest pain, or shortness of breath. There was no accompanying fever, chills, vaginal discharge, or bleeding. Physical examination revealed a non-tender, palpable left flank mass. Serology work-up revealed mild anemia, while tumor markers were within the normal range (Table 2).

**Table 2:** Tumor biomarkers are within the normal range CA 125: Cancer antigen 125; CEA: carcinoembryonic antigen; CA 19-9; carbohydrate antigen 19-9; LDH; lactate dehydrogenase; DHEA; dehydroepiandrosterone; AFP; alpha fetoprotein

Markers	Values
CA 125	15.3 IU/ml
CEA	0.7 ng/ml
CA 19-9	10.0 U/ml
LDH	132 IU/L
DHEA	252 ng/dl
AFP	2.6 ng/ml

A radiology work-up revealed a mixed cystic and solid mass with hemorrhage measuring 11.0 cm in the greatest dimension. No free fluid or hemorrhage was noted in abdominopelvic cavities, and other adnexal organs were unremarkable. Subsequently, the patient underwent laparoscopy, which showed a left ovarian mass with torsion. The external surface of the mass was smooth, and it was filled with thick brownish fluid. The intraoperative evaluation was consistent with adenocarcinoma, and the patient underwent exploratory laparotomy, total abdominal hysterectomy with bilateral pelvic and para-aortic lymph node dissection, and omentectomy.

A gross examination of the ovarian tissue showed multiple fragments of previously opened unilocular cysts with smooth lining, filled with brown malodorous content, along with a tan-white, firm necrotic mass. The external surface was smooth and shiny with an attached fallopian tube. The inner lining lacked papillary excrescences. Examination of the remaining organs was unremarkable. 

**Figure 1:**
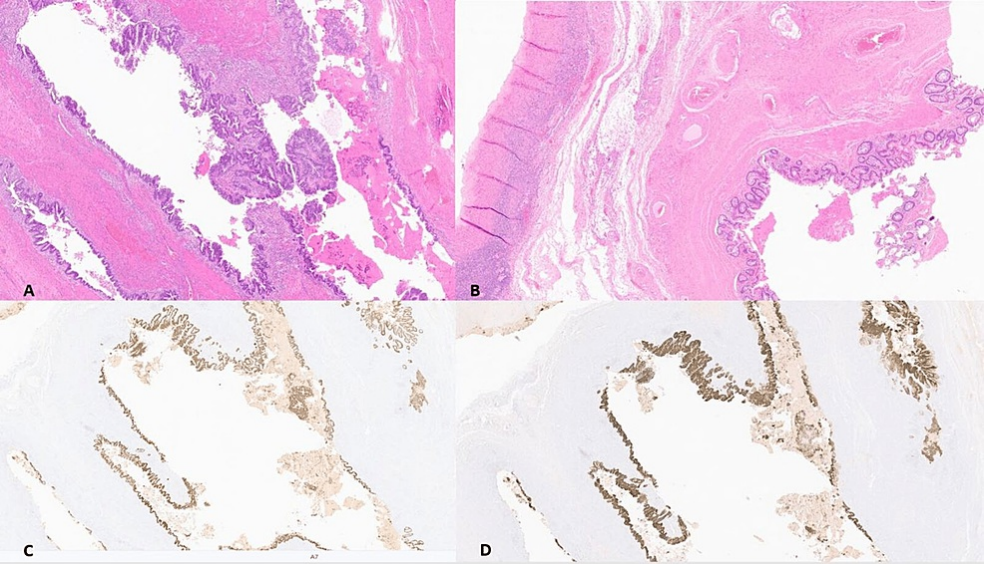
The teratoma showing malignant colorectal phenotype on H & E 100x (a). The teratoma showing benign epithelial lining of colorectum on H & E 40x (b). CDX2 and CK20 show strong positivity in malignant colorectal epithelium derived from MCT (c and d).

Extensive sampling of the ovarian cyst and mass was consistent with well to moderately differentiated gland-forming adenocarcinoma of colorectal phenotype with necrosis, arising from the epithelial lining of MCT (Figure 1A). Additional sections were submitted and benign colorectal epithelium arising from MCTs was also found (Figure 1B). For further classification of the lesion, immunohistochemistry (IHC) was done. IHC showed diffuse and strong positivity for CDX2 (Figure 1C) and CK20 (Figure 1D), while PAX8, CK7, estrogen receptor (ER), and progesterone receptor (PR) were negative. Morphology and IHC findings suggested colorectal-type somatic adenocarcinoma arising from mature cystic teratoma. Endoscopic and colonoscopy examinations were unremarkable, which made the tumor classified as stage IA ovarian cancer, and the patient was managed as such upon follow-up.

## Discussion

Teratomas are predominantly cystic and are commonly referred to as mature cystic teratomas or dermoid cysts. However, there are rare cases where teratomas can also appear as solid masses. They originate from totipotent stem cells and are typically found in the midline of the body. These totipotent stem cells can differentiate into various types of tissues, including ectodermal tissues such as skin and hair, mesodermal tissues such as muscle and bone, and endodermal tissues such as those found in the gastrointestinal tract and lungs.

The exact origin of teratomas is still not fully understood, but there are multiple theories regarding their development. Among these theories, the one that is widely accepted suggests that teratomas occur due to failures in meiosis II or meiosis I in premeiotic cells [23]. These failures in the genetic division process can lead to the formation of teratomas containing a wide range of differentiated tissues.

While teratomas are typically mature and benign, there are rare cases where malignant transformation can occur. The exact mechanism behind this transformation is not fully understood. However, it has been postulated that in the pelvic cavity, long-term exposure of MCTs to carcinogens may contribute to their malignant transformation [24].

The occurrence of somatic malignancies, particularly intestinal adenocarcinomas, within teratomas, is a rare event. These malignancies are believed to arise from the endodermal lining of the lower gastrointestinal tract. IHC studies have shown that these tumors exhibit positive staining for markers associated with intestinal differentiation, such as CDX2 and CK20. The presence of these markers supports the notion that the malignant component within the teratoma shares similarities with intestinal tissue in terms of cellular characteristics and protein expression [12].

Making a preoperative diagnosis of malignant transformation in teratomas is challenging. While certain factors, radiology findings, and serum markers can provide some clues, they are not definitive in confirming malignancy.

Factors such as old age, large tumor size (>10cm), postmenopausal status, and the presence of solid components within the teratoma can increase the likelihood of malignant transformation [22]. It is worth noting that despite these factors, many reported cases of malignant transformation have occurred in women of reproductive age [25]

Imaging modalities such as ultrasound and MRI can provide additional clues when evaluating teratomas for possible malignant transformation. On ultrasound, the presence of branching isoechoic components within the teratoma can raise suspicion for malignancy [26]. Similarly, on MRI, the identification of fat-suppression images can also suggest the possibility of malignancy within the teratoma.

In terms of serum markers, elevated levels of carcinoembryonic antigen and carbohydrate antigen 19.9 (CA 19.9) may raise suspicion for malignant transformation; however, they are not definitive diagnostic markers [26]. It is important to note that these markers can be elevated in various other malignancies and non-malignant conditions, so they lack specificity.

In cases where the malignant transformation is SCC, high levels of serum squamous cell carcinoma antigen (SCC-Ag) can be helpful. However, it should be noted that SCC-Ag levels are not particularly useful in cases of malignant transformation into intestinal adenocarcinomas and are not routinely used in clinical settings [27].

While these imaging findings and serum markers can provide some indications and raise suspicion, it is essential to consider them in the overall clinical context and in conjunction with other diagnostic tools. Ultimately, a definitive diagnosis of malignant transformation in teratomas relies heavily upon histopathological examination after surgical removal of the tumor.

A thorough sampling of the tissue, combined with a meticulous histopathological examination, is essential. The examination of morphology, as well as IHC markers such as CDX2 and CK20, can be highly valuable in diagnosing colorectal adenocarcinoma. However, it is worth noting that morphology and IHC alone cannot distinguish between somatic-type colorectal adenocarcinoma arising from MCTs and metastatic colorectal adenocarcinoma. It is crucial to differentiate between these two entities since it significantly impacts staging and management decisions. When colorectal adenocarcinoma is detected, it is important to conduct gastrointestinal screening to rule out the possibility of a primary tumor.

Lesions limited to the ovary typically do not require active treatment and can be managed through observation alone. However, in advanced stages, additional therapy is necessary. It is important to note that due to the rarity of such cases, there is currently no universally established chemotherapy regimen. Nevertheless, certain treatments based on platinum and 5-fluorouracil (5FU) are beneficial in some instances [7]. Additionally, KRAS mutations have been identified in certain cases, suggesting that targeted therapies directed at the epidermal growth factor receptor could potentially be effective [20].

Factors indicating a poor prognosis post-malignant transformation include the spread of the tumor, invasion of the cyst wall, the presence of ascites, spontaneous or accidental rupture of the tumor, adhesion to surrounding tissues, and certain tumor types other than squamous carcinomas. These factors are associated with a less favorable outcome and suggest a higher likelihood of aggressive behavior or disease progression [28].

## Conclusions

The preoperative diagnosis of malignant transformation presents significant challenges, leading to a complex diagnostic and therapeutic situation. The experience and expertise of pathologists can play a crucial role in enhancing intraoperative diagnosis. Furthermore, the final diagnosis of colorectal adenocarcinoma derived from MCTs as opposed to metastatic colorectal adenocarcinoma to the ovary has a momentous impact on clinical follow-up and therapy decisions. The rarity of this case is being highlighted to emphasize the importance of comprehensive sampling, careful examination of morphology, and the utilization of relevant IHC markers. Additionally, this case serves to underscore the fact that colorectal-type somatic adenocarcinoma can arise from MCTs due to malignant transformation.

## References

[1] Curling OM, Potsides PN, Hudson CN (1979). Malignant change in benign cystic teratoma of the ovary. Br J Obstet Gynaecol.

[2] Berek JS (2012). Berek & Novak’s Gynecology.

[3] Caruso PA, Marsh MR, Minkowitz S, Karten G (1971). An intense clinicopathologic study of 305 teratomas of the ovary. Cancer.

[4] Snir OL, DeJoseph M, Wong S, Buza N, Hui P (2017). Frequent homozygosity in both mature and immature ovarian teratomas: a shared genetic basis of tumorigenesis. Mod Pathol.

[5] Dalmau J, Gleichman AJ, Hughes EG (2008). Anti-NMDA-receptor encephalitis: case series and analysis of the effects of antibodies. Lancet Neurol.

[6] Ishiura H, Matsuda S, Higashihara M (2008). Response of anti-NMDA receptor encephalitis without tumor to immunotherapy including rituximab. Neurology.

[7] Ulker V, Numanoglu C, Akbayir O, Akyol A, Tuncel A, Akca A, Aydin O (2012). Malignant transformation arising from mature cystic teratoma of the ovary: a report of six cases. J Obstet Gynaecol Res.

[8] Kido A, Togashi K, Konishi I (1999). Dermoid cysts of the ovary with malignant transformation: MR appearance. AJR Am J Roentgenol.

[9] Christopherson WA, Councell RB (1989). Malignant degeneration of a mature ovarian teratoma. Int J Gynaecol Obstet.

[10] Templeman CL, Fallat ME, Lam AM, Perlman SE, Hertweck SP, O'Connor DM (2000). Managing mature cystic teratomas of the ovary. Obstet Gynecol Surv.

[11] Levine DA, Villella JA, Poynor EA, Soslow RA (2004). Gastrointestinal adenocarcinoma arising in a mature cystic teratoma of the ovary. Gynecol Oncol.

[12] Clark ME, Will MD (2016). Intestinal-type adenocarcinoma arising in a mature cystic teratoma of the ovary. Int J Gynecol Pathol.

[13] Fishman A, Edelstein E, Altaras M, Beyth Y, Bernheim J (1998). Adenocarcinoma arising from the gastrointestinal epithelium in benign cystic teratoma of the ovary. Gynecol Oncol.

[14] Ueda G, Fujita M, Ogawa H, Sawada M, Inoue M, Tanizawa O (1993). Adenocarcinoma in a benign cystic teratoma of the ovary: report of a case with a long survival period. Gynecol Oncol.

[15] Kushima M (2004). Adenocarcinoma arising from mature cystic teratoma of the ovary. Pathol Int.

[16] Güney M, Oral B, Demir F, Ozsoy M, Kapucuoğlu N (2006). Mucinous adenocarcinoma arising from the gastrointestinal epithelium in benign cystic teratoma of the ovary--case report. Eur J Gynaecol Oncol.

[17] Min KJ, Jee BC, Lee HS, Kim YB (2006). Intestinal adenocarcinoma arising in a mature cystic teratoma of the ovary: a case report. Pathol Res Pract.

[18] Takai M, Kanemura M, Kawaguchi H (2012). Mucinous adenocarcinoma of the intestinal type arising from mature cystic teratoma of the ovary: a rare case report and review of the literature. J Ovarian Res.

[19] Hershkovitz D, Vlodavsky E, Simon E, Ben-Izhak O (2013). KRAS mutation positive mucinous adenocarcinoma originating in mature ovarian teratoma: case report and review of literature. Pathol Int.

[20] Li Y, Zhang R, Pan D, Huang B, Weng M, Nie X (2014). KRAS mutation in adenocarcinoma of the gastrointestinal type arising from a mature cystic teratoma of the ovary. J Ovarian Res.

[21] Sunitha S, Arundhathi S, Anil M (2017). Adenocarcinoma of intestinal type arising in mature cystic teratoma of ovary in a young female: an incidental finding. Indian J Pathol Oncol.

[22] Wan KM, Foroughi F, Bansal R, Oehler MK (2019). Intestinal adenocarcinoma arising from a mature cystic teratoma. Case Rep Pathol.

[23] Caspi B, Lerner-Geva L, Dahan M, Chetrit A, Modan B, Hagay Z, Appelman Z (2003). A possible genetic factor in the pathogenesis of ovarian dermoid cysts. Gynecol Obstet Invest.

[24] Rim SY, Kim SM, Choi HS (2006). Malignant transformation of ovarian mature cystic teratoma. Int J Gynecol Cancer.

[25] Park JH, Whang SO, Song ES, Choi SJ, Lee WY (2008). An ovarian mucinous cystadenocarcinoma arising from mature cystic teratoma with para-aortic lymph node metastasis: a case report. J Gynecol Oncol.

[26] Mlikotic A, McPhaul L, Hansen GC, Sinow RM (2001). Significance of the solid component in predicting malignancy in ovarian cystic teratomas: diagnostic considerations. J Ultrasound Med.

[27] De Bruijn HW, Willemse PH, Ten Hoor KA, Boonstra H (1996). Raised serum squamous cell carcinoma antigen levels in malignant transformation of mature cystic ovarian teratoma. Int J Gynecol Cancer.

[28] Park CH, Jung MH, Ji YI (2015). Risk factors for malignant transformation of mature cystic teratoma. Obstet Gynecol Sci.

